# Ultrasound quantification of pleural effusion volume in supine position: comparison of three model formulae

**DOI:** 10.1186/s12890-024-03142-2

**Published:** 2024-07-04

**Authors:** Dachuan Tang, Huiming Yi, Wei Zhang

**Affiliations:** Department of Medical Ultrasound, Tongji Hospital, Tongji Medical College, Huazhong university of Science and Technology, Wuhan, Hubei 430030 China

**Keywords:** Pleural effusion, Ultrasound, Quantification, Volume

## Abstract

**Background:**

To investigate the accuracy of three model formulae for ultrasound quantification of pleural effusion (PE) volume in patients in supine position.

**Methods:**

A prospective study including 100 patients with thoracentesis and drainage of PE was conducted. Three model formulae (single section model, two section model and multi-section model) were used to calculate the PE volume. The correlation and consistency analyses between calculated volumes derived from three models and actual PE volume were performed.

**Results:**

PE volumes calculated by three models all showed significant linear correlations with actual PE volume in supine position (all *p* < 0.001). The reliability of multi-section model in predicting PE volume was significantly higher than that of single section model and slightly higher than that of two section model. When compared with actual drainage volume, the intra-class correlation coefficients (ICCs) of single section model, two section model and multi-section model were 0.72, 0.97 and 0.99, respectively. Significant consistency between calculated PE volumes by using two section model and multi-section model existed for full PE volume range (ICC 0.98).

**Conclusion:**

Based on the convenience and accuracy of ultrasound quantification of PE volume, two section model is recommended for pleural effusion assessment in routine clinic, though different model formulae can be selected according to clinical needs.

## Introduction

Pleural effusion (PE) is a common concurrent manifestation of multisystem diseases in clinical settings, associated with significant morbidity and mortality. Nonmalignant PE has been reported to lead to one-year mortality rates ranging from 25 to 57% [1]. Traditionally, CT imaging has been utilized to evaluate PE volume accurately through layer-by-layer analysis and three-dimensional reconstruction [2]. However, the high cost and radiation exposure associated with CT limit its frequent use in clinical practice. Consequently, ultrasound has emerged as a cost-effective, non-invasive alternative for quantifying PE.

Research efforts have been focused on developing ultrasound techniques for PE quantification for an extended period [2–6]. The objective is to formulate an equation capable of approximating the volume of PE using two-dimensional ultrasound images and their corresponding parameters [6, 7]. Despite the clinical variability in the significance of the exact volume of PE, due to maximum tolerable amounts ranging from 1000 to 1800 mL, accurate quantification is pivotal in scenarios requiring precise fluid management and therapeutic monitoring.

Recent advancements in medical imaging have enhanced the accuracy and reliability of pleural effusion quantification [8]. While computed tomography (CT) has traditionally been considered the gold standard due to its high resolution and three-dimensional volumetric analysis capabilities, its routine use is often limited by high costs and patient exposure to ionizing radiation [9]. In contrast, ultrasound provides a rapid, cost-effective, and radiation-free alternative. Although ultrasound relies on two-dimensional images, recent models have improved its accuracy through advanced algorithms and modeling techniques that estimate the three-dimensional volume of pleural effusions [7, 10, 11]. This study aims to validate the accuracy of these ultrasound-based models by comparing them not only to actual drainage volumes but also discussing their relative merits in comparison to CT imaging. Such comparisons are crucial for establishing ultrasound as a viable primary tool for pleural effusion assessment in clinical settings where CT availability may be constrained.

Furthermore, although chest radiographs are routinely used to assess post-procedural complications such as pneumothorax, they do not provide quantitative data essential for evaluating the efficacy of interventions aimed at reducing PE volume. This limitation underscores the clinical importance of ultrasound in providing detailed volumetric assessments that are critical for managing patient outcomes effectively. Therefore, this study seeks to determine the accuracy of single section, two section, and multi-section ultrasound models, using actual drainage volumes as a benchmark for comparison, thereby highlighting the potential of ultrasound to enhance clinical decision-making in the management of PE.

## Methods

This study prospective enrolled patients with PE at Tongji Hospital between February 2023 and June 2023. The inclusion criteria were as follows: (1) Age over 18 years old with stable vital signs. (2) Patients without history of thoracic surgery. (3) Patients without thoracic deformity. The exclusion criteria were as follows: (1) Patients with adverse reactions related to catheterization and drainage procedures. (3) Presence of a separation within the PE. (4) Poor ultrasound image acquisition leading to inaccurate data measurement. The study received approval from the Ethics Committee of Tongji Hospital, which is affiliated with Tongji Medical College, Huazhong University of Science and Technology. All participants submitted a written informed consent form.

### Ultrasound acquisition

**Fig. 1:**
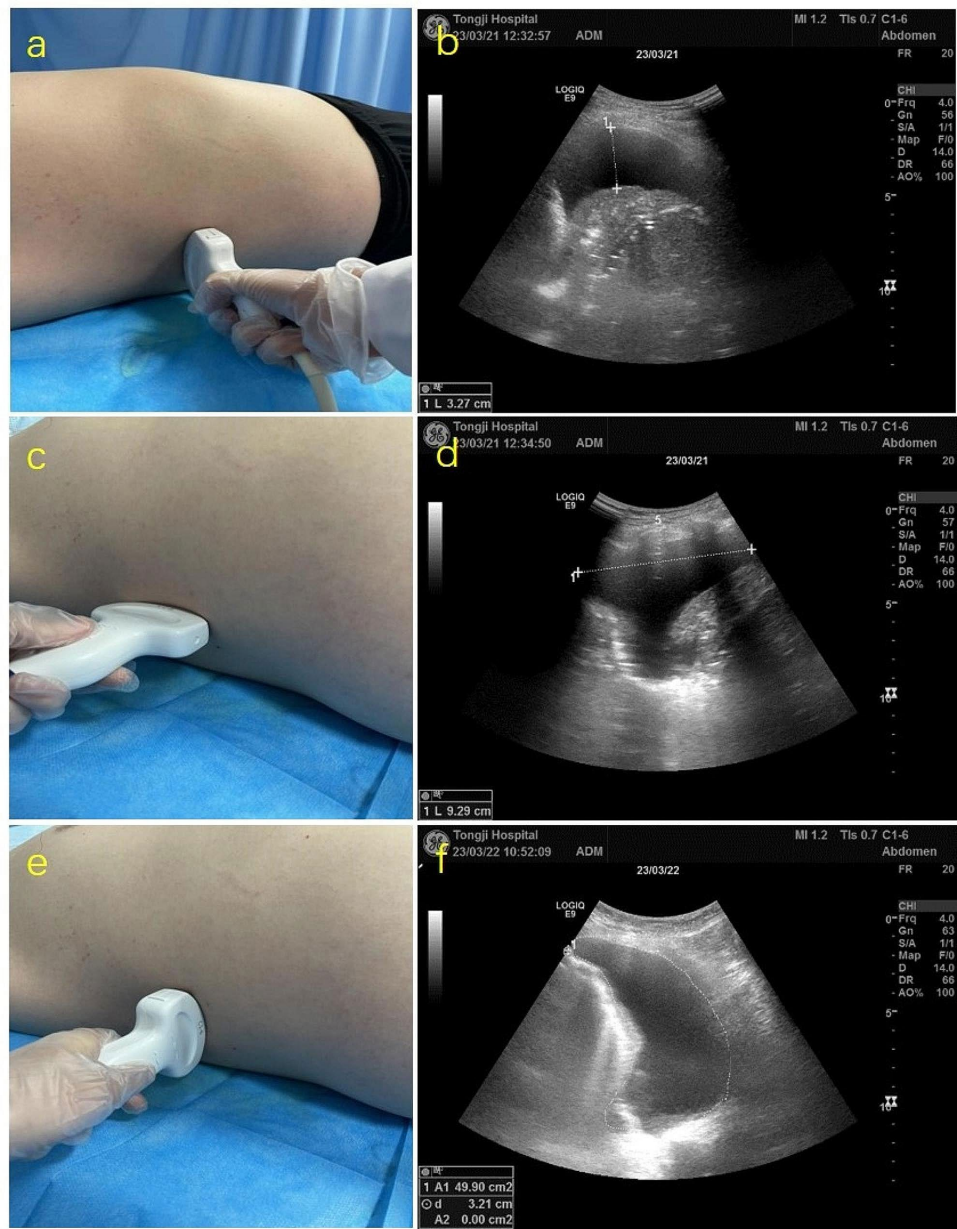
Illustration of ultrasound quantifications of pleural effusion volume. Patients were in supine position with a transducer placed at midaxillary line and perpendicular to chest wall. The maximum transverse distance between the lateral chest wall and the lung surface was recorded as Sep (mm) (**a** & **b**). The distance between upper point and bottom of the anechoic area in longitudinal section was recorded as maximum suprainferior length L (cm) (**c** & **d**). The anechoic area of each intercostal space was recorded as A (cm2) (**e** & **f**)

A LOGIQ E9 Ultrasound system (GE Healthcare, Little Chalfont, UK), equipped with a 3–5 MHz convex array transducer, was used for detailed ultrasound scanning and measurements prior to thoracentesis. Patients were positioned in a supine posture, breathing naturally. The transducer was placed on the midaxillary line and oriented perpendicular to the chest wall. Systematic scanning was conducted in transverse sections from one intercostal space to another, and in longitudinal sections laterally. This method allowed for the visualization of effusions, appearing as anechoic zones within the thoracic cavity. The maximum transverse distance between the lateral chest wall and the lung surface was recorded as Sep (mm). The distance from the uppermost to the bottom point of the anechoic area in the longitudinal section was recorded as the maximum suprainferior length (L in cm). The area of the effusion in each intercostal space was recorded as A (cm²) (see Fig. 1). Data were captured using end-expiratory frozen grey-scale ultrasound imaging, and the mean of three measurements was taken for each parameter.

### Thoracentesis and drainage

All patients underwent ultrasound-guided thoracentesis immediately after ultrasound data acquisition of PE. The procedures of thoracentesis and catheterization were performed by skilled radiologists or individuals possessing advanced expertise. The Seldinger catheterization procedure was used to indwell a 7 F pigtail tube (YB-A-II 2.3/235, Jiangsu Yubang Medical Equipment Technology Co., Ltd.) into the thoracic cavity, then a sterile bag was attached for drainage. The PE was drained continuously within 1 h, and ultrasound imaging showing no anechoic area between the visceral and parietal pleura was taken as evidence of complete drainage. The total amount of drainage was recorded as actual volume of PE when the drainage volume is less than 800 ml and ultrasound imaging showed complete drainage. If the drainage volume reached 800 ml and ultrasound still showed the presence of effusion in the pleural cavity, it is considered the actual PE volume was greater than 800 ml.

### Volume calculation formulae for different models

Single section model adopted the following formula for quantifying PE [4]:

1 $${V}_{single section}\left(ml\right)=20\times Sep \left(mm\right)$$

where V_single section_ was the calculated PE volume (ml), 20 was the correction factor, and Sep (mm) was the maximum vertical distance between the lateral chest wall and the lung surface.

Two section model simulated PE as a crescent-like cylinder with a crescent-like bottom, and its calculation method involved multiplying the maximum cranial length of the PE by the area of the anechoic zone at half the length. The specific formula was as follows [12]:

2 $${V}_{two section}\left(ml\right)=L\left(cm\right)\times {A}_{1/2L} \left({cm}^{2}\right)$$

where L represents the maximum cranial length, A_1/2L_ represented the area of the anechoic zone when the maximum cranial length was halved (1/2L). When the 1/2L point met with a rib, the average of the area between the upper and lower intercostal spaces at this point was denoted as A_1/2L_.

Multi-section model simulated PE as a combination of multiple crescent-like cylinders with gradual changes in size. The volume of this combination was the sum of the volumes of these crescent-like cylinders. The formulae were as follows [12]:

3 $${V}_{multi-section}\left(ml\right)=\sum _{1}^{n}{\varDelta V}_{n}{(cm}^{3})$$

4 $${\varDelta V}_{n}{ (cm}^{3})=\varDelta L\left(cm\right)\times {\varDelta A}_{n}{(cm}^{2})$$

5 $$\varDelta L\left(cm\right)=L\left(cm\right)/n$$

where ∆V_n_ represented the volume of each crescent-like cylinder, ∆A_n_ represented the area of the echogenic zone of each intercostal space, ∆L represented the length of each crescent-like cylinder, and n indicated the number of intercostal spaces involved by PE.

### Statistical analysis

All data were statistically analyzed using MedCal^®^ software and a *p*-value < 0.05 was regarded as statistically significant difference. The correlation analyses of PE volume derived from different models as well as actual drainage volume were performed using linear regression analysis, and Bland-Altman plots and Intra-class Correlation Coefficient (ICC) analyses were used to further confirm their correlation relationships. Among them, ICC < 0.4 indicates poor reliability, 0.4 ≤ ICC ≤ 0.75 indicates medium reliability, 0.75 < ICC < 0.9 indicates good reliability, and 0.9 ≤ ICC ≤ 1 indicates excellent reliability.

## Results

**Fig. 2:**
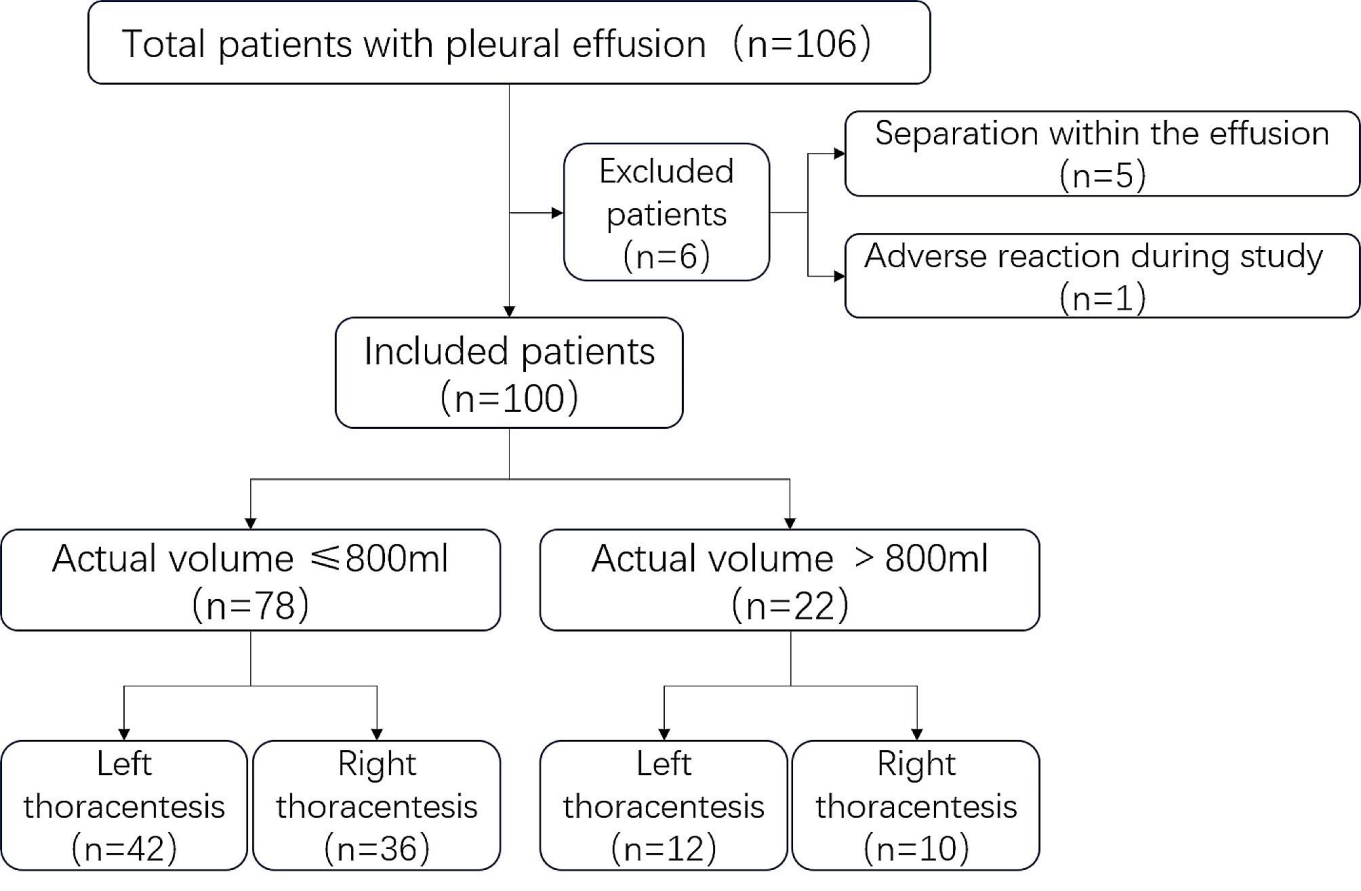
Flowchart of patient enrollment

100 patients (58 males and 42 females, mean age 65.4 years) with PE were included in this study. Based on the exclusion criteria, one patient who suffered from pleural reaction during the thoracentesis procedure was excluded. Additionally, five patients with encapsulated PE were also excluded. None were excluded due to poor ultrasound image acquisition (Fig. 2). In this study, each patient underwent left or right unilateral thoracentesis and drainage due to clinical needs. The characteristics of patients included were demonstrated in Table 1.

**Table 1:** Characteristics of patients included in this study

	Pleural effusion (PE) volume		total (*n* = 100)
	≤ 800 ml (*n* = 78)	> 800 ml (*n* = 22)	
Age (y)	54.7 ± 17.4	69.7 ± 11.1	65.4 ± 15.8
Male (%)	45 (57.7%)	13 (59.1%)	58 (58%)
Location			
Left PE	35 (44.9%)	7 (31.8%)	42 (42.0%)
Right PE	30 (38.5%)	8 (36.4%)	38 (38.0%)
Bilateral PE	13 (16.7%)	7 (31.8%)	20 (20.0%)
Etiology			
tumor-related	41 (52.6%)	10 (45.5%)	51 (51.0%)
infection-related	26 (33.3%)	8 (36.4%)	34 (34.0%)
polyserous effusion	8 (10.3%)	4 (18.2%)	12 (12.0%)
heart failure	3 (3.8%)	0	3 (3.0%)
Actual volume of pleural effusion (ml)	99.9 ~ 799.6^*^(530.2 ± 175.5)	838.6 ~ 1968.6^#^(1316.3 ± 333.7)	99.9 ~ 1968.6(703.1 ± 393.3)

Notes: *, drainage volume. #, calculated volume using multi-section model

**Fig. 3:**
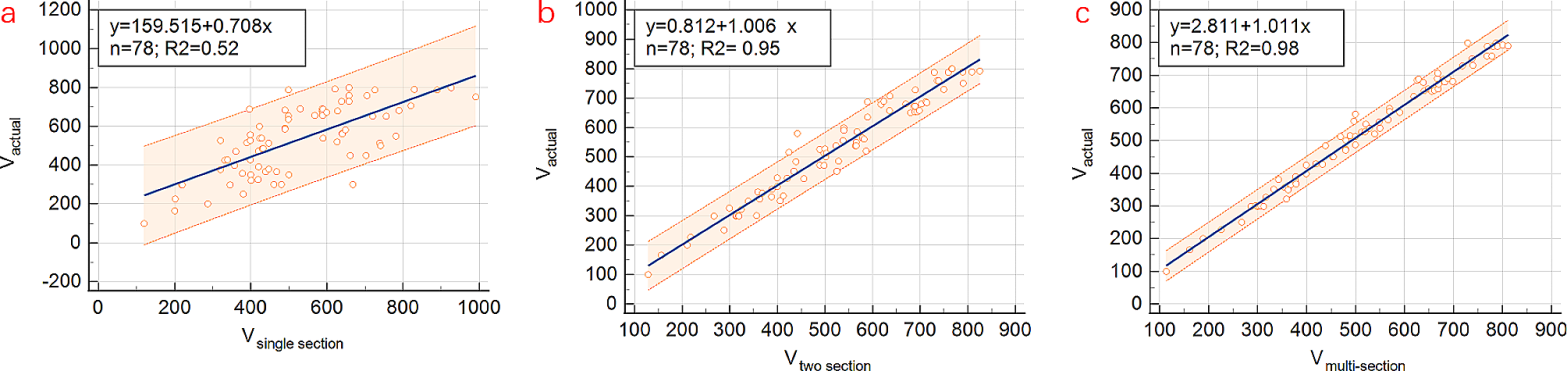
Regression analyses revealed significant linear correlations between actual drainage volume and calculated pleural effusion volumes derived from three model formulae

For patients with actual PE volume less than 800 ml, regression analyses revealed significant linear correlations between actual drainage volume and calculated PE volume derived from all three models, with *P* < 0.001 for all three models. The R2 values were found to be 0.52 for single section model, 0.95 for two section model, and 0.98 for multi-section model, respectively (Fig. 3). In the consistency test between actual PE volume and calculated volume using three models, the ICC of single section model, two section model, and multi-section model are 0.720, 0.972, and 0.992, respectively. Overall, the reliability of multi-section model is significantly higher than that of single section model and slightly higher than that of two section model (Table 2).

**Table 2:** Intra-class correlation coefficients (ICCs) among calculated volumes by three models and actual pleural effusion (PE) volume

Actual PE volume	Calculated PE volume comparison	ICC	95% CI
≤ 800 ml	single section model vs. actual drainage volume	0.72	0.59–0.81
	two section model vs. actual drainage volume	0.97	0.96–0.98
	multi-section model vs. actual drainage volume	0.99	0.99-1.00
> 800 ml	two section model vs. multi-section model	0.90	0.79–0.96
Full range	two section model vs. multi-section model	0.98	0.97–0.99

**Fig. 4:**
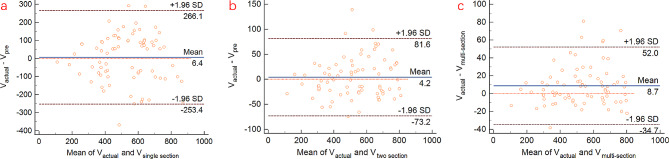
Bland-Altman scatterplot analyses revealed that most of the points of the three models fell within the 95% CI, indicating that the calculated volumes of the three models were consistent with actual pleural effusion volume

The Bland-Altman scatterplots (Fig. 4) revealed that most of the points of the three models fell within the 95% confidence interval (CI). This indicated that the calculated PE volumes of the three models were consistent with actual drainage volume. Notably, the 95% CI of two section model and multi-section model were smaller, signifying that the difference from actual drainage were smaller in these models. Moreover, the 95% CI of multi-section model was the smallest. Therefore, the Bland-Altman scatterplot demonstrated that the volume of PE calculated by multi-section model was in better agreement with actual PE volume, and multi-section model exhibited the highest reliability.

**Fig. 5:**
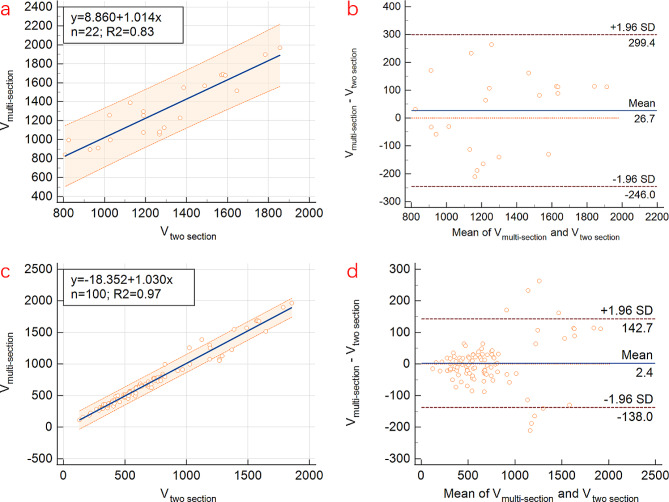
Linear regression and Bland-Altman scatterplot analyses showed significant correlation and consistency between calculated pleural effusion volumes by using two section model and multi-section model, both when actual volume > 800 ml (**a** & **b**) as well as when analyzing all patients together (**c** & **d**)

According to results demonstrated above, multi-section model can achieve robust prediction of actual PE volume. Therefore, aiming at avoiding the medical risk associated with actual drainage volume exceeding 800 ml, multi-section model was taken as an alternative tool to serve as a reference standard to analyze the value of two section model in estimating PE in large volume. In this study of patients with pleural volumes exceeding 800 ml, a comparison was made between the calculated pleural volumes using two section model and multi-section model. Linear regression analysis revealed a significant linear correlation between V_two section_ and V_multi−section_, with *P* < 0.001 and R2 of 0.83. The ICC was found to be 0.90, indicating a high level of agreement between these two models. Additionally, the Bland-Altman scatterplot analysis demonstrated that all data points fell within the 95% CI, further confirming the accuracy and consistency of two section model in estimating PE volume exceeding 800 ml (Fig. 5).

Furthermore, when all patients, including those with pleural volumes greater than 800 ml and those with volumes less than 800 ml, were analyzed together, it was found that V_two section_ is highly correlation with V_multi−section_, with a significant linear relationship (*p* < 0.001, R2 of 0.97) and ICC of 0.98. Additionally, the Bland-Altman scatterplot demonstrated that the majority of points fell within the 95% CI (Fig. 5). These results indicated that regardless of the volume of PE, two section model can robustly estimate the volume.

## Discussion

The innovative approach of this study lies in its detailed exploration of ultrasound techniques to estimate pleural effusion volume, particularly addressing inherent challenges such as respiratory movements. Respiratory diaphragmatic excursions during inspiration and expiration significantly affect the accuracy of ultrasound measurements of pleural effusion by altering the thoracic cavity dimensions and the distribution of fluid. To mitigate these variations, our methodology emphasized measurements during end-expiratory phases, where diaphragmatic movement is minimal, thereby enhancing measurement consistency and reliability. This approach not only improves the precision of pleural effusion quantification but also has substantial clinical implications. By providing reliable volume estimates, our study supports more precise management of pleural effusions, potentially guiding therapeutic decisions such as the need for drainage and monitoring treatment response, which are critical in clinical settings where accurate fluid assessment is paramount.

In clinic, thoracentesis and drainage are not suitable for all patients with PE according to etiology and PE volume [13]. Nevertheless, the evaluation of PE volume is essential for all patients during follow-up and monitoring post treatment [14]. Therefore, this study aimed to identify an imaging method capable of calculating the volume of PE accurately, conveniently and non-invasively. In this study, we circumvented the drawbacks associated with CT imaging and employed ultrasound measurements to predict the volume of PE by using three different model formulae. The results exhibited a high level of correlation and consistency between the calculated and actual volume of PE. Among the three models involved in this study, both two section model and multi-section model provided a more accurate result compared to single section model. Given the irregularity of PE morphology, the prediction accuracy of single section model is theoretically lower than that of two section model and multi-section model in volume estimation, and this study corroborates this assertion.

Both two section model and multi-section model of ultrasonic imaging showed good predictive performance, while multi-section model had the highest consistency with actual PE volume, indicating the highest measurement accuracy. Multi-section model decomposes the originally irregular space into multiple small volumes for quantitative addition, reducing the error of morphological standardization and making the results more accurate. However, in clinical applications, multi-section model requires more measurement data, and the operation and calculation are relatively more cumbersome and time-consuming. In contrast, although slightly lower consistency with actual PE volume existed, two section model can provide more convenient calculations and quicker predictions of PE volume.

Some previous studies have used CT as the gold standard for quantifying PE [15, 16]. However, it could be more reasonable to take actual drainage volume of the PE as a gold reference for the following reasons: Firstly, CT involves a measurement process that utilizes layer-by-layer manual boundary tracing and three-dimensional reconstruction. This process is influenced by the operator’s experience, introducing a degree of artificial measurement error [17]. Moreover, in daily clinical practice, there is often a time gap between CT examination and ultrasound examination. This time gap may lead to changes in PE volume due to the complexity of the patient’s condition. In this study, chest drainage was performed immediately after ultrasound data acquisition, and actual drainage volume served as the gold standard for PE volume. This approach maximized the comparability between ultrasound measurement and actual PE volume. Previous studies assessing PE volume through ultrasound approach were primarily conducted with patients in a seated position [18, 19]. However, in clinic, seated position cannot be maintained for many patients, such as critically ill, immobile, or comatose patients. In this study, all patients were scanned in the supine position, making the results more widely applicable to clinical scenarios. Previously, Scarlata, S., et al. [12], quantified PE volume in seated patients using stereoscopic models, which demonstrated high consistency with CT quantification (ICC 0.971). In contrast, taking actual PE drainage volume as the gold standard, this study performed ultrasound measurements with patients in the supine position and showed that two section model accurately assessed PE volume (ICC 0.974). These findings were consistent with the results of Scarlata S, indicating that two section model can accurately estimate actual volume of PE both in sitting and supine positions. Therefore, two section model should be recommended with top priority for PE assessment in daily clinic practices in terms of convenience and accuracy, though clinicians may prefer different models according to specific needs,

In this study, we acknowledged the potential variability in ultrasound measurements that can arise from differences in probe positioning and operator technique. To mitigate these factors, we implemented standardized procedures across all examinations. Each ultrasound scan was performed using a LOGIQ E9 system with a 3-5MHZ convex array transducer, strictly following a protocol that included specific instructions for probe placement and angulation. The probe was consistently positioned at the midaxillary line and maintained perpendicular to the chest wall to ensure uniformity in the acoustic window and measurement angles. Furthermore, all sonographers involved in this study were experienced radiologists who had undergone specific training to standardize their scanning technique for this project, focusing on maintaining consistency in the depth and angle of view. These measures were designed to reduce the influence of inter-operator variability and enhance the reliability of our volumetric assessments of pleural effusion.

In addressing the factors identified by the reviewer, it is important to consider that patient body habitus, presence of underlying lung diseases, and positioning during ultrasound measurements can significantly influence the accuracy of pleural effusion volume estimations. Body habitus, including variations in chest wall thickness and adiposity, can alter the acoustic window and penetration of the ultrasound beam, potentially leading to suboptimal image quality and measurement discrepancies. Similarly, underlying lung diseases such as pulmonary fibrosis or emphysema may complicate the interpretation of ultrasound images due to altered lung architecture and the presence of additional echogenic structures. Furthermore, the positioning of patients, particularly those unable to maintain a supine posture due to discomfort or respiratory distress, can affect the distribution and visualization of pleural effusion. Systematic adjustments for these variables are crucial for enhancing the precision of ultrasound-based volumetric assessments. Efforts to standardize patient positioning, optimize transducer placement, and adjust for individual anatomical and pathological variations are essential for improving the reliability of these measurements in clinical practice.

There are some limitations in this study. First, the sample size is limited and the results may only apply to cases included in this study. In the future, a multi-center study with large sample size should be conducted to verify the accuracy of different model formulae. Second, actual PE volume verified in this study was limited to patients with a PE volume of ≤ 800 ml. In clinical practice, the maximum drainage of one-set PE does not typically exceed 800 ml to prevent the occurrence of recurrent pulmonary edema. Therefore, the quantitative predictive performance of different models for large PE volume (> 800 ml) has not been verified in this study. Additionally, this study did not validate the quantification of encapsulated PE. The results of this study may not be applicable to encapsulated PE due to the greater variation in their morphology and the difficulty of draining them in one go. Furthermore, the application of ultrasound for PE quantification is subject to a steep learning curve, and the accuracy of the measurements is significantly dependent on the operator’s expertise. This factor introduces variability, as less experienced operators might not achieve the same level of precision as their more experienced counterparts. Moreover, the inherent limitations of 2D ultrasound mean that it may not fully capture the three-dimensional nature of pleural effusions, potentially affecting the accuracy of volume measurements. Finally, this study requires a high level of accuracy in data acquisition, so skilled and experienced radiologists are required to perform the measurements.

Our study, constrained by an 800 mL threshold for pleural effusion volume due to clinical protocols aimed at preventing re-expansion pulmonary edema, highlights a limitation with the reliance on single field-of-view ultrasound measurements. Addressing cases where the maximum cranial length exceeds this view, future work should explore integrating multiple ultrasound scans and adjusting protocols for larger effusions. Advancements such as three-dimensional ultrasound, MRI, or enhanced CT scans, combined with sophisticated algorithms, promise significant improvements in measuring complex effusions, potentially enhancing clinical decisions and treatment strategies. Moreover, developing automated systems could minimize operator variability, expanding ultrasound’s robustness and applicability in clinical practice. Future research must extend beyond current limitations to incorporate larger effusion volumes and integrate advanced imaging technologies, thus enhancing diagnostic precision and clinical utility.

## Conclusion

Pleural effusion volumes calculated by three models (single section model, two section model and multi-section model) all showed significant linear correlations with actual PE volume in supine position. The reliability of multi-section model in predicting PE volume was significantly higher than that of single section model and slightly higher than that of two section model. Significant consistency between calculated PE volumes by using two section model and multi-section model existed for full PE volume range. Given the convenience of ultrasound measurement and the accuracy of the results, two section model is recommended for pleural effusion assessment in routine clinic, though different model formulae can be selected according to clinical needs.

## Data Availability

The datasets used and analyzed during this study are available from the corresponding author on reasonable request.
